# Dysbiosis–NK Cell Crosstalk in Pancreatic Cancer: Toward a Unified Biomarker Signature for Improved Clinical Outcomes

**DOI:** 10.3390/ijms26020730

**Published:** 2025-01-16

**Authors:** Sara Fanijavadi, Lars Henrik Jensen

**Affiliations:** 1 Cancer Polyclinic, Levanger Hospital, 7601 Levanger, Trøndelag, Norway; 2 Department of Oncology, Vejle Hospital, University Hospital of Southern Denmark, 7100 Vejle, Denmark; lars.henrik.jensen@rsyd.dk; 3 Department of Oncology, Institute of Regional Health Research, University of Southern Denmark, 7100 Vejle, Denmark

**Keywords:** pancreatic cancer, dysbiosis, NK cell, biomarkers, treatment resistance, side effects

## Abstract

Pancreatic ductal adenocarcinoma (PDAC) is an aggressive cancer with poor prognosis, primarily due to its immunosuppressive tumor microenvironment (TME), which contributes to treatment resistance. Recent research shows that the microbiome, including microbial communities in the oral cavity, gut, bile duct, and intratumoral environments, plays a key role in PDAC development, with microbial imbalances (dysbiosis) promoting inflammation, cancer progression, therapy resistance, and treatment side effects. Microbial metabolites can also affect immune cells, especially natural killer (NK) cells, which are vital for tumor surveillance, therapy response and treatment-related side effects. Dysbiosis can affect NK cell function, leading to resistance and side effects. We propose that a combined biomarker approach, integrating microbiome composition and NK cell profiles, can help predict treatment resistance and side effects, enabling more personalized therapies. This review examines how dysbiosis contributes to NK cell dysfunction in PDAC and discusses strategies (e.g., antibiotics, probiotics, vaccines) to modulate the microbiome and enhance NK cell function. Targeting dysbiosis could modulate NK cell activity, improve the effectiveness of PDAC treatments, and reduce side effects. However, further research is needed to develop unified NK cell–microbiome interaction-based biomarkers for more precise and effective patient outcomes.

## 1. Introduction

Pancreatic ductal adenocarcinoma (PDAC) is the third deadliest cancer in high-income countries, with poor prognosis mainly due to its dense tumor microenvironment (TME) and immunosuppressive niche, which promote tumor progression and treatment resistance [1,2,3]. The TME also provides a favorable environment for microbial survival, leading to dysbiosis that induces immune suppression, including natural killer (NK) cell dysfunction [4]. Currently, no universal screening method exists for early PDAC detection or predicting treatment responses [5]. Moreover, there are limited diagnostic tools for assessing cancer treatment side effects, underscoring the need for new strategies.

Microbial communities, including bacteria, fungi, and viruses, play crucial roles in both health and disease [6,7]. Dysbiosis—an imbalance in these communities—has been linked to various diseases, often involving pathogenic overgrowth, loss of beneficial microbes, or reduced microbial diversity. While defining a healthy microbiota is challenging due to technical limitations, studying the mechanisms of dysbiosis provides important insights into disease processes [8].

Several studies characterized the tumor microbiota in PDAC, emphasizing the microbiome’s role in PDAC development, progression, and treatment response. Microbiota from the oral, gut, bile duct, and tumor sites influence tumor biology by inducing genetic mutations, reshaping the immune landscape, and regulating cancer metabolism and oncogenic pathways [9,10,11,12]. Research on microbiome differences between PDAC patients and healthy individuals has led to investigations on how microbial communities interact in the oral, gut, bile duct and tumor niches to affect PDAC outcomes [13,14,15,16]. Metagenomic, metaproteomic, and metabolomic analyses of microbiota samples have revealed that intratumoral microbiota diversity correlates with survival outcomes. Higher microbial diversity has been associated with better overall survival in resected PDAC patients. Specific microbes such as Megasphaera and Sphingomonas are linked to longer survival, and Clostridium with shorter survival [14,15,16,17]. These findings suggest that microbiome composition could serve as a prognostic biomarker for PDAC. Microbial species, particularly from the phyla Firmicutes and Proteobacteria, are predominant in PDAC, with Proteobacteria being most abundant in pancreatic tumors and associated with metastasis [18,19,20].

The gut microbiota also influences immune responses. Specific bacteria like *Citrobacter freundii* and *Shigella sonnei* have been shown to upregulate oncogenic pathways and contribute to immunosuppression, reprogramming the TME. Furthermore, *Acidovorax ebreus* (*A. ebreus*) is linked to reduced levels of immune cells, further promoting PDAC progression. Metabolites converted by the microbiota can regulate immune cell accumulation and activation within tumors, affecting oncogenic pathways, reducing immune cell-mediated cytotoxicity [18,19,21]. Interestingly, studies in other cancer types have shown that intratumoral microbial injections, such as *Propionibacterium acnes*, can accelerate tumor growth by increasing inflammatory cytokines like TNF-*α* and IL-1*β*, which activate oncogenic signaling pathways, including the Hedgehog pathway [22,23]. Since the Hedgehog pathway is a major driver in PDAC, affecting immune surveillance, further research is needed to understand how intratumoral microbes interact with these pathways in PDAC.

NK cells are crucial in immune surveillance and tumor control, targeting drug-resistant cancer cells and eliminating malignant cells without relying on major histocompatibility complex (MHC) or tumor-associated antigen presentation [24,25]. They rely on a balance of activating and inhibitory receptor signals to execute cytotoxic functions, including antibody-dependent cell-mediated cytotoxicity (ADCC). NK cells can also interact with dendritic cells (DCs) to initiate adaptive immune responses, enhancing tumor immunity [26,27,28]. However, NK cell dysfunction due to cancer or treatments can impact therapy efficacy and side effects. In PDAC, where NK cells are rare in the tumor tissue and show reduced cytotoxic activity [29], targeting NK cell activity is an emerging strategy for improving therapeutic outcomes.

Recent findings suggest that the gut microbiota plays a crucial role in promoting NK cell infiltration into tumors [26]. For example, in a mouse tumor model colonized with *Helicobacter hepaticus*, improved cancer prognosis was observed, along with an increased number of NK cells within the tumor [30]. Additionally, microbiota-mediated metabolite conversion regulates NK cell cytotoxicity, further enhancing the antitumor immune response. However, PDAC patients often exhibit a CD16^hi^CD57^hi^ phenotype of peripheral NK cells, with reduced cytotoxic activity, low IFN-*γ*, and high IL-10 levels, contributing to immune evasion. Tumor cells also limit NK cell movement to the tumors, further impairing their antitumor function [29,31].

Furthermore, clinical studies, including a clinical trial (NCT05462496), are investigating the modulation of the gut microbiome with pembrolizumab following chemotherapy in resectable PDAC patients. This interventional trial aims to evaluate how altering the gut microbiota may influence immune response and treatment outcomes in PDAC. Notably, another study indicates that in PDAC patients positive for Enterobacter and Enterobacteriaceae in the tumor or TME, the immune receptor TIGIT is significantly upregulated in NK cells. This upregulation has been associated with sustained clinical benefits, suggesting that the gut microbiota can modulate NK cell proliferation and function by influencing immune checkpoint receptors like TIGIT [32,33]. However, TIGIT acts as an inhibitory receptor in T cells [32]. This distinction underscores the importance of context in understanding TIGIT’s role across different immune cell types.

Boosting NK cells in PDAC treatment is a promising area of research. Tumor cells often block NK cell migration to the tumor, reducing their anti-tumor effects. Targeting these pathways may be crucial in enhancing NK cell responses in PDAC. While strategies like NK-cell-recruiting antibodies and engineered nanogels have shown potential, they have limitations. Therefore, targeting the microbiome to modulate NK cell activity may be a more practical and effective approach than direct NK cell therapies.

In addition to their role in cancer development, microorganisms also influence treatment-related side effects. Several studies have explored the role of microorganisms in the development of oral lesions and mucositis, a common side effect of cancer treatments [34,35,36].

This review explores how dysbiosis contributes to NK cell dysfunction in the PDAC TME and highlights the significance of both tumor-infiltrating and peripheral NK cells in shaping immune responses. We hypothesize that microbial metabolites from dysbiosis suppress NK cell activity, promoting tumor progression and resistance to therapies. We also suggest that dysbiosis–NK cell interactions may contribute to treatment side effects. Targeting dysbiosis could restore NK cell function, improve therapeutic outcomes, and reduce side effects. This review will examine current evidence supporting these hypotheses and discuss strategies for microbiome manipulation as a novel approach to improving PDAC treatment.

## 2. Interaction Between Microbiota and NK Cell Function in PDAC

**Figure 1:**
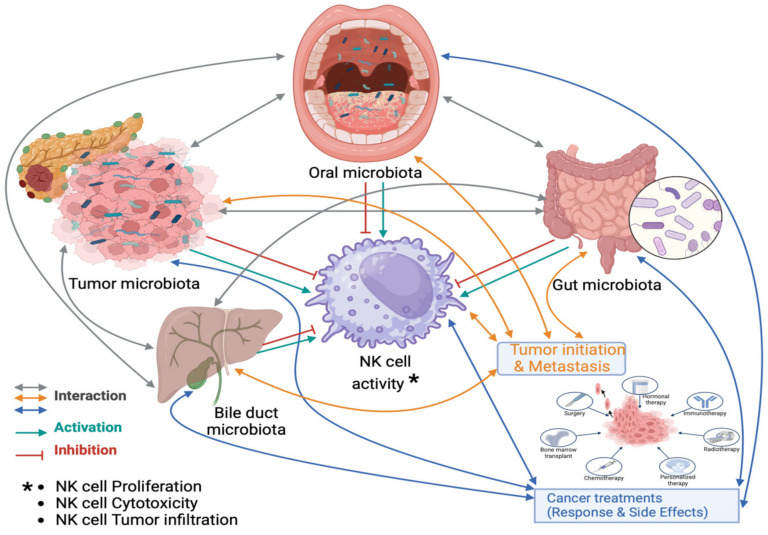
Microbiota–NK cell interaction and PDAC clinical outcome. This figure illustrates the complex interactions between different microbiota (oral, gut, and tumor-associated) and their crosstalk with NK cells in pancreatic ductal adenocarcinoma (PDAC). The microbiota communities not only interact with NK cells but also influence each other; oral microbiota can alter the gut and, ultimately, the tumor-associated microbiota, while tumor-associated microbiota can also modify the gut and oral microbiota. Dysbiosis, a pathological disruption of the microbiota community, can suppress NK cell function, including reducing NK cell frequency, cytotoxicity, and tumor infiltration. In contrast, a balanced microbiota can enhance NK cell activity, promoting tumor surveillance and immune response. Dysbiosis, however, contributes to tumor initiation, progression, resistance to treatment by impairing NK cell-mediated immune responses, and side effects of cancer treatments.

The interaction between the microbiota and NK cell function is important for understanding the progression of PDAC and treatment resistance (Figure 1). Clinically, analyzing buccal swabs and saliva, which contain leukocytes, including NK cells, in varying proportions, can provide insights into this interaction [37,38]. Additionally, using stool samples to detect microbiota can serve as a surrogate marker for NK cell activity in patients. These samples help us understand the relationship between microbiota, NK cell function, and PDAC, including their impact on responses to cancer treatment and associated side effects [39,40,41,42,43,44,45,46,47].

### 2.1. Dysbiosis-Induced NK Cell Dysfunction in PDAC

The microbiota profiles can differ significantly based on cancer type [48]. In pancreatic cancer, notable changes in both oral and intrapancreatic microbiota have been observed. Oral dysbiosis is characterized by an altered abundance of microorganisms such as *Porphyromonas gingivalis*, Fusobacterium, *Neisseria elongata*, *Streptococcus mitis*, Bacteroides, Leptotrichia, *Grabulitacetlla adiacens*, and *Aggregatibacter actinomycetemcomitans*, while the intrapancreatic microbiota shows shifts in the levels of Gammaproteobacteria, Fusobacterium, *Escherichia coli*, and *Bifidobacterium pseudolongum* [10,17,19,49]. Additionally, the gut microbiota plays a crucial role in influencing the effectiveness of anti-cancer treatments and can significantly affect the quality of life of patients undergoing cancer therapy [40]. Therefore, the microbiome holds potential as a non-invasive tool for early cancer detection.

**Figure 2:**
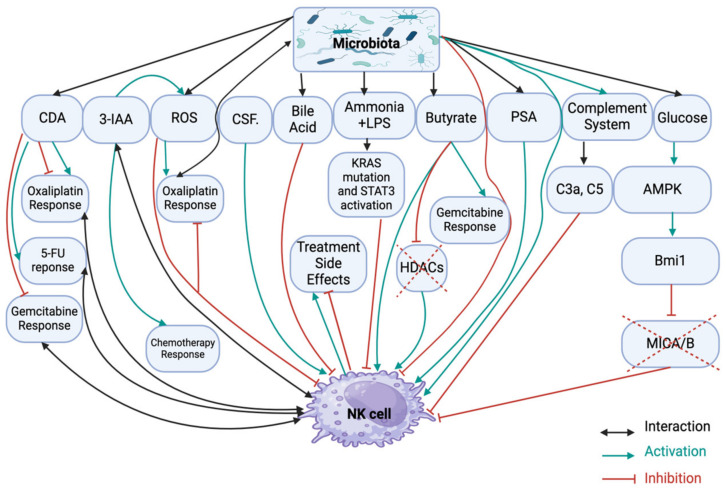
Microbiota–NK cell interactions and their impact on clinical outcomes. This figure illustrates the various mechanisms through which the microbiota can influence NK cell activity and treatment outcomes in pancreatic ductal adenocarcinoma (PDAC). The diagram highlights the complex interplay between microbiota, NK cells, and chemotherapy. Specifically, it focuses on the AMPK-Bmi1-GATA2-MICA/B pathway, which plays a critical role in tumor immune evasion. Under conditions of energy stress, such as high glucose, AMPK is activated, leading to the upregulation of Bmi1. Bmi1, in turn, activates GATA2, which reduces the expression of MICA/B ligands. These ligands normally stimulate NK cells to recognize and attack tumor cells. By downregulating MICA/B, this pathway helps cancer cells evade NK cell-mediated immune surveillance, thereby promoting tumor growth and resistance to treatment like 5-fluorouracil (5-FU). Gut microbiota metabolites can be broadly categorized into three types based on their origin: (1) metabolites produced directly by the microbiota from dietary components, such as short-chain fatty acids (SCFAs like butyrate) and indole derivatives (like IAA), which play key roles in gut health and immune modulation (via inhibition of histone deacetylases (HDACs)); (2) metabolites generated by the host and modified by the microbiota, like secondary bile acids, which influence metabolism and immune functions; and (3) metabolites produced de novo by the microbiota, such as polysaccharide A (PSA), which helps maintain immune balance. Additionally, some products like uridine, produced by cytidine deaminase (CDA) enzyme, can also be assumed as de novo metabolites. These metabolites, along with other postbiotics like cell-free components (CSF), exert various effects on the host, including modulating inflammation and gut integrity.

Many studies have investigated the role of microbiota in the initiation and progression of PDAC [38,40,48,50], with some suggesting NK cell suppression as a key factor [44,45,46]. However, the role of dysbiosis and its interaction with NK cells remains underexplored (Figure 2).

NK cells play a key role in the early immune response to bacterial, viral, and fungal infections by activating macrophages, dendritic cells, and neutrophils before the adaptive immune system is triggered [41,45,46]. While Toll-like receptors (TLRs) have been extensively studied in macrophages, recent research underscores their critical role in NK cells as part of the first-line defense against pathogens. TLRs on NK cells help these cells recognize and respond to infections, contributing to the body’s early defense mechanisms before the adaptive immune system becomes active. Different TLRs are expressed on NK cells, with TLR1 showing the highest expression, followed by TLR2, TLR3, TLR5, and TLR6. TLR ligands can activate NK cells directly or indirectly, though TLR9 expression is typically low or undetectable [41,42,43]. Commensal microbiota instruct nonmucosal mononuclear phagocytes to prime NK cells. The NK cell-activating receptor NKp44 has been observed to directly interact with bacteria from the genus Mycobacterium, *Nocardia farcinica*, and *Pseudomonas aeruginosa* [42,43,44,45]. Microbiota-derived stimulator of interferon genes (STING) agonists stimulate intratumoral monocytes to produce type I interferon (IFN-I), influencing macrophage polarization and enhancing NK cell–DC communication. These findings suggest a microbiota-driven mechanism in the TME with potential implications for optimizing cancer therapies [44,45,46]. *Lactobacillus plantarum* effectively enhances the expression of the natural cytotoxicity receptor (NCR) family and IL-22 in NK cells [47]. *A. muciniphila* has been found to boost immune function by modulating specific cytokines and interacting with TLRs, including TLR2 and TLR4. This bacterium also plays a protective role against pathogens, notably reducing inflammation triggered by *Porphyromonas gingivalis*, an opportunistic pathogen associated with periodontitis, which is implicated in the development of PDAC [46,47,48,49]. A preclinical study shows that diet change and gut microbiota affect the growth of PDAC by influencing NK cells. Microbiota-derived substances can boost NK cells’ ability to fight tumors. This suggests that using gut microbiota to support the immune system could help treat PDAC and improve survival rates [39]. Another preclinical study revealed that Pseudoxanthomonas, Saccharopolyspora, and Streptomyces were associated with increased NK cell infiltration in colorectal cancer, whereas *Anabaena* sp. K119, an uncultured bacterium, *P. putida* str. KT2440, *T. chromogena*, and *E. rectale/Roseburia* showed a negative relationship with NK cell infiltration [49]. Further investigation revealed that extracts from *B. pseudolongum* could modify tumor-associated macrophages (TAMs), increasing tolerogenic cytokines such as IL-10 through TLR signaling activation. TLRs are crucial for the activation of innate immunity, as they identify specific patterns in microbial components. Previous studies have indicated that the polarization of macrophages impacts the activity of NK cells in PDAC [43,51].

Pathogenic components of *Helicobacter pylori*, such as ammonia, lipopolysaccharide (LPS), and inflammatory cytokines, contribute to pancreatic damage and carcinogenesis by activating NF-*κ*B and AP-1 signaling, leading to *KRAS* mutations and persistent STAT3 activation, which all contribute to NK cell suppression [52,53,54].

Investigating how microbial imbalances affect NK cell function could reveal novel therapeutic targets and enhance the efficacy of existing treatments. By elucidating these interactions, we can develop more effective approaches to managing PDAC and ultimately improve patient outcomes.

### 2.2. Dysbiosis-Induced Drug Resistance in PDAC

Tumor microbiome communities are significantly different between PDAC patients with long-term survival and short-term survival [15,16]. Gut microbiota and tumor-associated microbes affect the anticancer therapies, highlighting their potential as novel targets for future interventions. The permeability of the intestinal barrier may be a cause of bacterial dissemination into the pancreatic duct, which requires further validation. Gut microbes are generally believed to influence tumor microbiome composition through three primary routes: direct transfer via the pancreatic duct, spread through the lymphatic system, and translocation through the porto-mesenteric circulation [6]. Intratumoral microbes could affect tumor susceptibility to chemotherapy and patient outcome [55,56,57,58].

Studies show that neoadjuvant chemotherapy and pre-operative biliary stenting significantly alter the microbiota composition, which can impact treatment responses and post-operative complications in PDAC [57,58,59,60,61,62]. Studies have shown that gut bacteria play a crucial role in the effectiveness of PD-1-based immunotherapy [63], with dysbiosis linked to impaired immune cell function and reduced treatment response. In contrast, a balanced microbiome, particularly the presence of beneficial bacteria like Bifidobacterium, has been shown to enhance the efficacy of immunotherapy [64,65,66,67].

Many studies focus on gemcitabine–microbiome interaction. For example, *E. coli* influences the effectiveness of gemcitabine by promoting resistance and activating tumor cytotoxicity [66]. Gammaproteobacteria metabolize gemcitabine, inducing drug resistance via the bacterial enzyme cytidine deaminase (CDA), while CDA conversely directs 5- Fluorouracil (5-FU) to tumor cells, reducing its toxic effects and improving its pharmacokinetic properties. Interestingly, the gene encoding CDA has been identified as one of the top upregulated metabolic genes in tumors that are resistant to immunotherapy. High CDA expression in pancreatic cancer cells is associated with resistance to DNA-damaging agents. Inhibition of CDA, both in patient-derived primary cultures and orthotopic xenografts, increased replication stress and enhanced the sensitivity of pancreatic adenocarcinoma cells to oxaliplatin. The exact effects of CDA on NK cells are not fully known, but its role in nucleotide metabolism, immune regulation, and inflammation suggests it could influence NK cell activity [67,68,69,70].

Butyrate is a controversial substance in cancer therapy due to its complex role in treatment resistance. While gemcitabine alters the “intestinal” microbiota to produce butyrate, which can trigger inflammation, some studies suggest that intratumoral butyrate contributes to chemoresistance in colorectal cancer (CRC). Butyrate’s effects vary depending on its location, with its influence on the TME distinct from its actions in peripheral circulation [42]. Despite this, many recent studies have explored butyrate’s potential antitumor effects, especially in enhancing innate immune responses. However, most research has focused on CRC, so its effects on other cancer types remain unclear [71,72,73].

Platinum-based chemotherapy relies on the generation of reactive oxygen species (ROS) to exert its cytotoxic effects. The gut microbiome can enhance ROS production induced by oxaliplatin, potentially improving treatment effectiveness. However, secondary resistance to platinum-based chemotherapy may arise from interactions between ROS and NK cell activity. ROS can alter the surface charge of NK cells, making it more difficult for them to adhere to and kill cancer cells, which may reduce their effectiveness and contribute to resistance [74,75].

Hyperglycemia-induced dysbiosis can impair the response to chemotherapy drugs like gemcitabine and paclitaxel [76]. High glucose levels promote the proliferation and epithelial–mesenchymal transition of PDAC cells, while also reducing NK cell activity against these cells through the AMPK-Bmi1-GATA2-MICA/B pathway [77]. This suggests that elevated glucose helps create an immunosuppressive tumor microenvironment. PDAC tumor cells further evade IFN-I signaling, suppressing innate immune responses [78]. Metformin, an anti-hyperglycemic drug, has been shown to activate NK cells in PDAC by inhibiting AKT phosphorylation, which triggers the STING/IRF3/IFN-*β* pathway. This activation enhances immune responses and may improve the effectiveness of cancer treatment, potentially by modifying the duodenal microbiome [79,80].

Recent studies emphasize the need to expand microbiome research to include viruses, helminths, fungi, protozoa, and archaea, in addition to bacteria [81]. For example, fungal community has also been shown to influence the efficacy of gemcitabine-based chemotherapy. In vivo depletion of the mycobiome using antifungal treatments was found to enhance the response of orthotopic PDAC tumors to chemotherapy [82]. Malassezia fungi can suppress NK cell activity by activating the complement system, which releases molecules like C3a and C5a that reduce NK cell cytotoxicity. Chronic inflammation from complement activation may also recruit immune cells that suppress NK cells. Additionally, Malassezia may lower the levels of NKG2D receptors, supporting PDAC growth [82,83]. Archaea, a distinct domain, can influence the host through their metabolites and contribute to cancer. Specifically, methanogenic archaea produce methane and can affect NK cell function by modulating dendritic cells [81]. Although not studied in PDAC, their potential impact on NK cells underscores the need for further research on archaeal metabolites in PDAC diagnosis and treatment. Further studies are needed to investigate NKA and its association with treatment resistance, particularly in the context of microbiota and NK cell interactions in PDAC.

### 2.3. Dysbiosis-Induced Treatment Side Effects

Recent studies have highlighted the potential of microbiota as a diagnostic tool for PDAC, but the prediction of treatment side effects remains less explored [15,84]. Microbial dysbiosis could serve as an important marker for identifying side effects of cancer treatments [36]. Microbial communities in the oral cavity, gut, and tumor play a critical role in influencing the immune system and modulating responses to cancer therapies. Alterations in these microbial communities, known as dysbiosis, may serve as early indicators of treatment efficacy, prognosis, and potential side effects of cancer therapies [84,85,86]. The buccal mucosa and oral microbiota are the first point of exposure to environmental factors such as food, drinks, and medications [87]. Similarly, the gut mucosa can also be directed by medication like Irinotecan, a chemotherapy agent used in PDAC and CRC, which disrupts gut microbiota, increasing harmful bacteria and causing toxicity. Its active metabolite, SN-38, is inactivated in the liver to SN-38 glucuronide and excreted into the intestine, where bacterial β-glucuronidase reactivates it into a toxic form, leading to the gastrointestinal canal, and potential systemic infection [36] Changes in the oral microbiota can trigger genetic and epigenetic changes in the buccal mucosa, potentially influencing the behavior of immune cells like NK cells, which are crucial in determining clinical outcomes during cancer treatment [88]. Dysbiosis in the oral microbiota may also lead to altered sensitivity of the oral mucosa to certain cancer treatments, contributing to side effects such as stomatitis (after 5-FU) or neuropathy (after oxaliplatin). Interestingly, some patients experience these side effects while others do not, suggesting that variations in the oral microbiota could predict who will develop these adverse effects [89,90].

Importantly, the oral microbiota is interconnected with both the gut and tumor microbiota. Changes in the oral microbiota can influence gut microbiota, which may, in turn, impact the tumor microenvironment. Gut microbiota composition has been linked to gastrointestinal side effects in patients treated with drugs such as irinotecan and 5-FU [89,90,91]. However, the presence or absence of these symptoms varies among patients, reinforcing the idea that microbiome composition may predict the risk of treatment-related side effects. Emerging evidence suggests that the gut microbiome and its interactions with the innate immune system play a significant role in gastrointestinal side effect development. The immune system can influence microbiome composition, while the microbiome, in turn, affects immune cell function [92]. Understanding these bidirectional interactions may improve risk prediction, personalize treatment strategies, and lead to targeted interventions to reduce or prevent chemotherapy-induced side effects in the future.

Current PDAC treatments, such as gemcitabine with nab-paclitaxel (nano albumin-bound paclitaxel) or FOLFIRINOX (a combination of 5-fluorouracil, leucovorin, irinotecan, and oxaliplatin), show objective response rates of 20–40% [93,94]. However, both oxaliplatin and nab-paclitaxel can cause neuropathy, which often leads to treatment discontinuation [95]. These chemotherapeutic agents trigger inflammatory factors that affect neurons and glial cells, contributing to the development of nociceptive pain in neuropathy [96]. 16S rDNA sequencing of rats with paclitaxel-induced peripheral neuropathy (PIPN) showed shifts in gut microbiota, with an increase in Bacteroides and UCG-005 genera and a reduction in Turicibacter, *Clostridium sensu stricto* 1, and Corynebacterium [97]. Despite extensive research on preventing oxaliplatin-induced neuropathy (OIPN), no definitive intervention has been proven effective. Most studies have focused on pharmacological treatments, while non-pharmacological approaches remain underexplored. More research is needed, particularly into alternative strategies such as microbiota modulation and modifying NK cell phenotypes, which could offer new solutions for mitigating OIPN [98]. Some studies have linked the occurrence of chemotherapy-induced neuropathy to changes in NK cell function.

NK cells, which are essential for immune surveillance, utilize several mechanisms to induce cytotoxicity. Key among these is the CD95/FasL system, TRAIL receptors, and NKG2D receptors on NK cells, which interact with ligands on stressed, tumor, or infected cells. However, NK cell activity is also regulated by inhibitory signals from MHC class I molecules, which can prevent the destruction of healthy cells [29,44,45].

## 3. Targeting Dysbiosis in PDAC for Overcoming Treatment Resistance and Managing Side Effects

**Figure 3:**
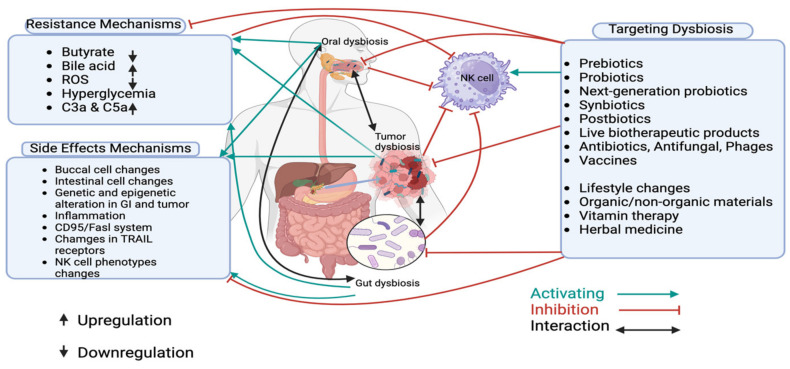
Dysbiosis and targeting dysbiosis in PDAC. This figure summarizes the mechanisms of treatment resistance and side effects in pancreatic ductal adenocarcinoma (PDAC), focusing on the role of microbiota and NK cells. It illustrates how microbiota influence NK cell activity, contributing to both tumor immune evasion and resistance to therapy. Additionally, the figure highlights how microbiota dysbiosis can worsen treatment side effects, such as inflammation and gastrointestinal toxicity. Non-invasive strategies, including dietary changes, probiotics, and fecal microbiota transplantation (FMT), are shown as potential methods to restore microbiome balance, improve therapeutic response, and reduce adverse effects in PDAC.

Targeting dysbiosis in PDAC presents a promising strategy for improving treatment outcomes (Figure 3). Microbiome modulation can be achieved either directly—through interventions such as antibiotics or probiotics to alter microbial communities—or indirectly by modifying lifestyle factors, using organic/non-organic materials, vitamin therapy or herbal medicine [99,100,101,102,103,104,105,106,107,108].

Microbiome-based strategies have been increasingly explored in managing cancer treatment resistance and alleviating chemotherapy side effects [109,110,111]. These strategies aim to improve therapeutic outcomes by enhancing immune responses, particularly NK cell activation [112]. While lifestyle modifications, such as high salt or the use of antioxidant vitamins and supplements, have been suggested as non-invasive ways to modulate the microbiome and activate NK cells [39,113], this section focuses specifically on direct microbiome-targeting approaches. These include prebiotics, postbiotics, and antibiotics, which can modify the microbiome and enhance NK cell function to improve PDAC management.

### 3.1. Prebiotics

Prebiotics are nutrients broken down by gut microbiota, impacting both the intestinal microenvironment and other organs. Prebiotics nourish probiotics and stimulate various functions in the host [114]. Many studies confirm the direct and indirect effect of prebiotics on the mediators of the immune response, including NK cells [115]. A preclinical study revealed that some genes involved in PDAC tumor progression and inflammatory response were downregulated in a group of mice fed with a high level of prebiotic-resistant starch diet compared to a control group [116]. Preclinical and clinical studies on the effects of prebiotics on NKA in PDAC and their correlation with overall survival would be extremely valuable, revealing potential therapeutic targets.

### 3.2. Probiotics

Several studies have shown that probiotics can reduce pancreaticoduodenectomy complications and inhibit cancer cell growth, although some research highlights potential risks, such as sepsis, due to the movement of probiotics in critically ill patients [114,117]. Many studies discussed the effects of probiotics on gastrointestinal cancers [118] and confirmed that Lactococcus lactis, a probiotic bacterium found in yogurt and cheese, can decrease various inflammatory agents that contribute to cancer, including both anti-inflammatory and pro-inflammatory molecules such as interleukin (IL)-6, IL-18, TNF-*α*, and NK cells [119,120]. In individuals with NK suppression, consuming fermented milk containing *Lactobacillus casei* strain Shirota and *Lactobacillus rhamnosus* HN001 has significantly increased NKA, aiding in cancer prevention [121]. Ferrichrome, derived from the probiotic strain Lactobacillus casei ATCC334, has shown potential for combating PDAC [122].

Probiotics can aid in the eradication of *H. pylori* or in the management of *H. pylori*-related diseases through various mechanisms, such as enhancing the strength of the mucosal barrier, competing for adhesion sites, and modulating the immune response [123]. The role of NK cells in the local immune response to *H. pylori* infection has been discussed [124]. Ongoing research is exploring the role of NK cells activated by probiotics in enhancing the adaptive immune response against pathogens [125].

A meta-analysis suggested probiotics as a potential preventive measure for immune checkpoint inhibitor-induced diarrhea, especially in cases where the severity reaches grade ≥ 2 [126].

A randomized controlled trial found that probiotic supplementation may help reduce chemotherapy-induced cognitive impairment in breast cancer patients by decreasing synaptic damage, oxidative stress, and glial cell activation in the central nervous system [127]. Similarly, probiotics have shown promise in enhancing chemotherapy outcomes in PDAC. In a PDAC xenograft mouse model, the use of probiotics not only improved the effectiveness of gemcitabine but also helped mitigate chemotherapy-induced toxicity [128].

Furthermore, Bifidobacterium potentiates the efficacy of immune checkpoint inhibitor [129]. Oxaliplatin and cisplatin demonstrate enhanced efficacy in the presence of healthy microbiota [130].

Focusing on NK cell activation in PDAC patients is crucial, and employing probiotics may offer a safe and effective strategy to achieve this.

### 3.3. Next-Generation Probiotics

Based on comparative microbiota analyses, next-generation probiotics (NGPs) are live microorganisms that, when administered in sufficient amounts, provide health benefits to the host [131]. NGPs are identified using bioinformatics and next-generation sequencing. They meet novel food regulations for safety and toxicity and have clear modes of action. Unlike traditional probiotics, NGPs include diverse microbial species, target specific diseases, and can be used as biotherapeutics [132].

For example, using *Faecalibacterium prausnitzii* has shown promise in enhancing PDAC treatment strategies [21]. Another example is selenium-enriched Bifidobacterium longum, a type of next-generation probiotic that is a species of bacteria that belongs to the genus Bifidobacterium. This probiotic may help mitigate Irinotecan-induced hepatotoxicity, reducing the inflammatory response by lowering levels of pro-inflammatory cytokines such as IL-1*β* and IL-18, while also promoting the expression of tight-junction proteins like occludin and ZO-1 [133].

A lower abundance of *Akkermansia muciniphila* causes multiple diseases, including cancer, in both mouse models and humans [134]. The use of Akkermansia as a representative example of NGPs needs further investigation.

### 3.4. Synbiotics

Synbiotics are combinations of prebiotics and probiotics designed to enhance the health of humans or animals [114]. *Bifidobacterium lactis* (*B. lactis* HN019) and *Lactobacillus rhamnosus* HN001 (*L. rhamnosus* HN001) have exhibited pronounced immunity-enhancing effects, particularly when combined with oligosaccharide-rich substrates, in both animal and human studies [135].

A recent study recruited ninety PDAC patients and randomly assigned them to one of three groups: placebo, probiotics, or synbiotics. Treatments were administered for 14 days pre-surgery and one-month post-surgery. The synbiotics group showed a significant increase in IFN-*γ* and a significant decrease in inflammatory cytokines compared to the other groups, suggesting that synbiotics enhance treatment efficacy and reduce side effects [136]. Interestingly, many studies have already suggested synbiotics as a promising adjunctive therapy for alleviating chemotherapy-associated symptoms in patients with solid tumors [137].

Emphasizing NK cell activation in PDAC through further clinical studies is essential, and utilizing synbiotics could offer a safe and effective strategy for achieving this.

### 3.5. Postbiotics

Postbiotics are beneficial substances like short-chain fatty acids (SCFAs), exopolysaccharides, vitamins, phenols, bacterial lysates, supernatants, enzymes, and cell wall fragments derived from the gut microbiota [138]. Studies have shown that postbiotics could play a role in preventing and treating gastrointestinal cancer [139]. Although they can prevent cancer, postbiotics exhibit selective toxicity, targeting only certain tumor types [140].

The cell-free supernatant from *Faecalibacterium prausnitzii* A2-165 promoted the increase in anti-inflammatory cytokines (IL-10, TGF-*β*2, and IL-1Ra) and reduced levels of key pro-inflammatory cytokines like IL-6, TNF-*α*, and TNF-*β* [141].

3-indoleacetic acid (3-IAA) is also a type of postbiotic, produced by certain gut bacteria, and can influence various physiological processes. Tintelnot et al. revealed that the microbiota-derived metabolite 3-IAA significantly enhances the effectiveness of chemotherapy in treating PDAC [120]. Investigating whether 3-IAA can activate NK cells is an emerging area of research.

Butyrate is a postbiotic produced by gut bacteria during fiber fermentation, offering various health benefits. In PDAC, butyrate has been shown to boost gemcitabine-induced apoptosis in cancer cells while also preserving intestinal mucosal integrity by reducing the abundance of pro-inflammatory microorganisms [71]. Supplementing conventional chemotherapy with butyrate or SCFA-producing bacteria, such as *Faecalibacterium prausnitzii*, may enhance clinical outcomes in PDAC [71,73,141] Interestingly, butyrate accumulates in higher concentrations and inhibits histone deacetylases, which are known to suppress NK cell activity, thereby enhancing its anticancer effects [142,143,144].

Further investigation into the application of postbiotics in the management of PDAC, particularly in combination with chemotherapy, is a very promising area of research.

### 3.6. Live Biotherapeutic Products

Live biotherapeutic products (LBPs) are products that contain live organisms used to prevent, treat, or cure human diseases. Unlike vaccines, they do not function by directly stimulating an immune response but rather by interacting with the body’s microbiota or other biological systems [129,145]. The effectiveness of LBPs can be influenced by various environmental factors, including diet [146]. LBPs can range from the transplantation of entire microbial communities to the introduction of individual bacterial strains, either engineered or non-engineered.

#### 3.6.1. Fecal Microbiota Transplantation

Fecal microbiota transplantation (FMT) is being explored as a therapeutic strategy to modulate the microbiota and treat dysbiosis, with potential benefits for cancer treatment outcomes and a reduction in treatment-related side effects [147,148].

A preclinical study found that transferring fecal material from PDAC-bearing mice (but not from healthy controls) accelerated tumor progression, highlighting the complexity of microbiome interactions in cancer [149].

A study by Riquelme et al. showed that transferring stool from long-term PDAC survivors (LTSs) into mice with advanced PDAC reduced tumor growth compared to stool from short-term survivors (STSs) or healthy controls. The LTS stool also increased immune markers like IFN-γ and IL-2, suggesting a stronger NK cell function and immune response. This study identified a protective bacterial signature in the stools of LTS PDAC patients, showing a dynamic relationship between the gut microbiome and PDAC progression [15].

Notably, FMT has been studied for its potential to modulate the gut microbiome and improve responses to immunotherapy in various solid tumors. For instance, a phase I trial (NCT03772899) evaluated the safety and efficacy of FMT combined with anti-PD-1 immunotherapy in patients with advanced melanoma, achieving a 65% objective response rate, including complete responses in some cases (20%). This promising result could be attributed to the indirect activation of NK cells by anti-PD-1 therapy, which mitigates NK cell exhaustion. While this trial did not focus on PDAC, the potential contribution to the observed effects cannot be excluded, suggesting that microbiome-targeted therapies combined with NK cell activation could be a promising approach. However, there are ongoing early-phase clinical trials investigating the combination of FMT and immunotherapy in PDAC to evaluate the safety and feasibility (NCT04975217).

FMT has also shown promise in managing infections and side effects related to cancer treatment. For example, FMT can help manage *Pseudomonas aeruginosa* infections resulting from chemotherapy-induced gut dysbiosis, such as 5-fluorouracil-induced mucositis [148]. Additionally, FMT using Lachnospiraceae and Roseburia species has been shown to reshape the gut microbiota, potentially alleviating chemotherapy-induced diarrhea [150]. Furthermore, FMT was found to reduce chemotherapy-induced peripheral neuropathy (PIPN) in rats by modulating astrocyte function and interfering with the TLR4/p38MAPK signaling pathway, suggesting its potential in managing neurological side effects [97].

It has also shown promise in reducing the adverse effects of immunotherapy and improving the efficacy of immune checkpoint inhibitors [150,151].

Given these promising findings, further research into FMT from long-term PDAC survivors or chemotherapy/immunotherapy responders—particularly its impact on NK cell activity and clinical outcomes—is crucial for fully understanding its therapeutic potential.

#### 3.6.2. Tumor-Colonizing Bacteria

Tumor-colonizing bacteria, a type of probiotic, prefer hypoxic environments and specific metabolites, allowing them to target tumors over healthy tissues [146]. Salmonella, Listeria, and Clostridium target tumors because they thrive in hypoxic environments and utilize specific metabolites [152,153,154]. These bacteria can kill tumor cells directly or stimulate the immune system. For example, LPS triggers immune responses, and Listeria spp. increase IL-12 production, enhancing anti-tumor effects via NK cell responses [153], and *Salmonella typhimurium* inhibits tumor growth by inducing the pro-inflammatory cytokine interleukin-1*β* [154].

Another study showed that the efficacy of anti-PD-1/PD-L1 treatment in gastrointestinal cancer could be enhanced by SCFA-producing bacteria, such as Eubacterium, Lactobacillus, and Streptococcus [155].

Intratumoral pseudoxanthomas have been shown to be more prevalent in PDAC patients with long overall survival [15], suggesting that increasing their levels through tumor-colonizing probiotics could potentially improve clinical outcomes.

Further studies are needed to develop tumor-colonizing bacteria as effective NK cell stimulators in the management of PDAC. However, their effectiveness relies on successfully colonizing tumors and metastases, which can be challenging in certain tissues.

### 3.7. Antibiotics, Antifungal and Phage Therapy

In human PDAC, bacteria can metabolize gemcitabine, with this effect linked to intratumoral LPS. This can be countered using antibiotics [66]. Research in PDAC mouse models has shown that the gut and tumor microbiomes are distinct, and antibiotic treatment can alter the tumor microenvironment, boosting immune surveillance and enhancing responsiveness to immunotherapy [51,154,155,156,157].

Many studies have also discussed that depleting the gut microbiome with antibiotics can make PDAC tumors more sensitive to chemotherapy [158,159,160]. Supporting this, an independent retrospective study found that patients treated with antibiotics during gemcitabine-containing regimens had higher survival rates, suggesting that correcting dysbiosis with antibiotics could help mitigate treatment resistance [158].

However, antibiotics can also have negative effects. For example, Iida et al. found that antibiotics reduced the effectiveness of oxaliplatin and cisplatin [130]. Treatment with vancomycin decreased Firmicutes and increased Proteobacteria, leading to higher bile acid levels, which can promote PDAC carcinogenesis [160]. Various antibiotic approaches need to be investigated to deplete tumor-favoring bacteria or treat dysbiosis to enhance the treatment outcomes of PDAC. On the other hand, antifungal drugs have shown potential in improving PDAC treatment [161], with Malassezia identified as a possible target [162].

In addition to antibiotics and antifungal therapy, phage therapy is emerging as a promising option. Phages are viruses that specifically target bacteria, with minimal impact on the surrounding microbiome. They could be used to target harmful bacteria or deliver drugs directly to tumors [163,164]. However, more research is needed to overcome the current limitations of phage therapy.

### 3.8. Vaccines and Oncolytic Viruses (OVs)

Research suggests that a healthy gut microbiome may improve the effectiveness of immune therapies, including vaccines and oncolytic viruses (OVs), by boosting NK cell activity [31,40]. Some studies indicate that certain microbes can influence NK cells, which could impact the success of cancer vaccines [165]. For example, Gopalakrishnan et al. showed that the gut microbiome can modulate the efficacy of checkpoint inhibitors by influencing NK cells and other immune cells, suggesting that microbiome-related factors could also influence vaccine responses in PDAC treatment [166].

Many studies explore different ways to improve dendritic cell vaccines by using NK cell interactions and offer an overview of important NK cell factors for immune monitoring [165]. Better NK cell activity, in turn, could lead to a stronger immune response to vaccines, potentially improving treatment outcomes in PDAC. Further research into how specific microbiota or gut-derived signals affect NK cell responses to vaccines in PDAC would be valuable in optimizing therapeutic strategies.

Clinical trials have explored vaccines in the management of PDAC, with some showing promising results. For instance, a Phase I clinical trial demonstrated anti-angiogenic activity of a prime-boost vaccination regimen with VXM01, an oral T cell vaccine targeting VEGFR2, in patients with advanced PDAC. An extension of the study, conducted three years later, confirmed the continued safety of the vaccine [167,168].

Additionally, a phase II trial with 90 PDAC patients found that CRS-207 plus cyclophosphamide or the GVAX vaccine was safe. However, these combinations did not improve OS compared to standard chemotherapy [169,170].

OVs are viruses, either genetically engineered or naturally derived, that specifically kill tumor cells while sparing normal ones. OVs and NK cells share a synergistic relationship in anticancer therapy [31,171]. OVs, with their ability to kill tumors and boost immunity, can overcome these challenges and enhance PDAC treatment when used in combination therapies [31]. Vesicular Stomatitis Virus (VSV), a type of OV, shows promise in early clinical trials. While effective against many PDAC cell lines, some resist VSV. Overcoming this resistance involves reducing antiviral defenses and combining VSV with other treatments like NK cell augmenting approaches [171].

Interestingly, some microbial communities can alter viral replication or clearance, potentially affecting the success of OVs in treating cancer [172]. Incorporating microbiome research into vaccine and OV development could enhance the therapeutic potential of these modalities, especially in terms of their ability to activate NK cells and strengthen the immune response in PDAC.

## 4. Discussion and Future Directions

Recent studies highlight the critical role of microbiome dysbiosis in PDAC, influencing clinical outcomes. Dysbiosis, characterized by microbial imbalances in the oral cavity, gut, bile duct, tumor, and its microenvironment, is linked to PDAC progression. Specific bacteria, such as *Porphyromonas gingivalis* and *Aggregatibacter actinomycetemcomitans*, are associated with increased PDAC risk [15,16,49]. Additionally, microbial metabolites from *Gammaproteobacteria* can impair chemotherapy efficacy, contributing to drug resistance [15,67].

Interestingly, while Lactobacillus species are typically considered probiotics, recent research by Matsukawa suggests that their enrichment in PDAC might have a potential carcinogenic role, particularly in the TME [6]. This raises the question: Which specific Lactobacillus species are enriched in the PDAC gut microbiome? Given the complexity of Lactobacillus strains, it can be suggested to distinguish between these strains and other microbial communities, as well as to understand their differential roles in PDAC progression.

Most studies analyze microbiome data at the phylum, order, or genus level, but species- and strain-level profiling is probably essential for more precise insights. For instance, *Listeria welshimeri* (Serovar 6B SLCC 5334) has been shown to exhibit unique properties that could influence disease progression in PDAC, providing more detailed information than genus-level data. This underscores the importance of focusing on species- and strain-specific markers in PDAC management [21,145,153,169]. Future research should prioritize such detailed microbiome analyses to develop more accurate microbial signatures for PDAC treatment. However, it can be argued that it is unlikely for a single species or strain to be solely responsible for disease progression in PDAC. In contrast, studying the balance of the entire microbial community may offer a more accurate representation of its role in disease progression. This idea is supported by the effectiveness of community-level interventions, such as FMT, which have shown greater success in other disease compared to individual probiotics [148]. These suggestions indicate that targeting the entire microbial community, rather than single organisms, may also be an effective approach.

Although oral microbiome analysis remains underexplored, a study by Kartal et al. found no significant differences in taxa abundance within the salivary microbiome but suggested stool microbiome signatures for PDAC [147]. Given the ease of saliva collection, further research into the oral microbiome is needed, and improving collection methods could provide valuable insights. Additionally, Nagata et al. observed differences between the gut and oral microbiomes in PDAC [85], suggesting that these compartments, along with the intratumoral microbiome, should be studied simultaneously to better understand their dynamic interactions and role in PDAC mechanisms.

Notably, dysbiosis has also been shown to disrupt NK cell function, which is essential for immune surveillance and combating PDAC [26,27,28,29,30,39,41,42,43,44,45,46]. However, the relationship between the microbiota and NK cells varies across different cancers. For example, while pathogenic bacteria such as *Fusobacterium nucleatum* suppress NK cell cytotoxicity in CRC [119], the dense fibrotic stroma in PDAC creates a barrier, amplifying microbiome-induced suppression of NK cells. In CRC, the TME is more accessible to microbial influence, enabling both suppression and activation of NK cells. Similarly, in lung cancer, gut microbiota modulation has been linked to enhanced NK cell activity and better immunotherapy outcomes, such as improved responses to immune checkpoint inhibitors [141]. In contrast, the microbiome and TME in PDAC predominantly fosters an immunosuppressive environment, limiting NK cell activation and reducing treatment efficacy. This underscores the necessity for NK cell augmentation therapies in PDAC [172]. In gastric cancer, interactions between *Helicobacter pylori* and NK cells also highlight immune evasion strategies, but unlike PDAC, gastric cancer often involves a single dominant microbe rather than the diverse dysbiotic communities seen in PDAC [122]. These differences highlight the need for cancer-specific approaches to understand and target microbiota–NK cell interactions.

Another important consideration is that, while our discussion focuses on the microbiome’s role in PDAC, studies in other diseases, such as Primary Sclerosing Cholangitis (PSC), have also moved toward therapeutic microbiome interventions. For example, the PSC-VANCO and FARGO trial protocols are based on mechanistic studies and microbiome modulation in PSC [173,174]. These studies could provide useful insights for PDAC, particularly in exploring therapeutic interventions that target the microbiome as a whole rather than individual microbes. However, these interventions are still in the early stages, and more research is needed to determine their relevance and applicability to PDAC.

**Figure 4:**
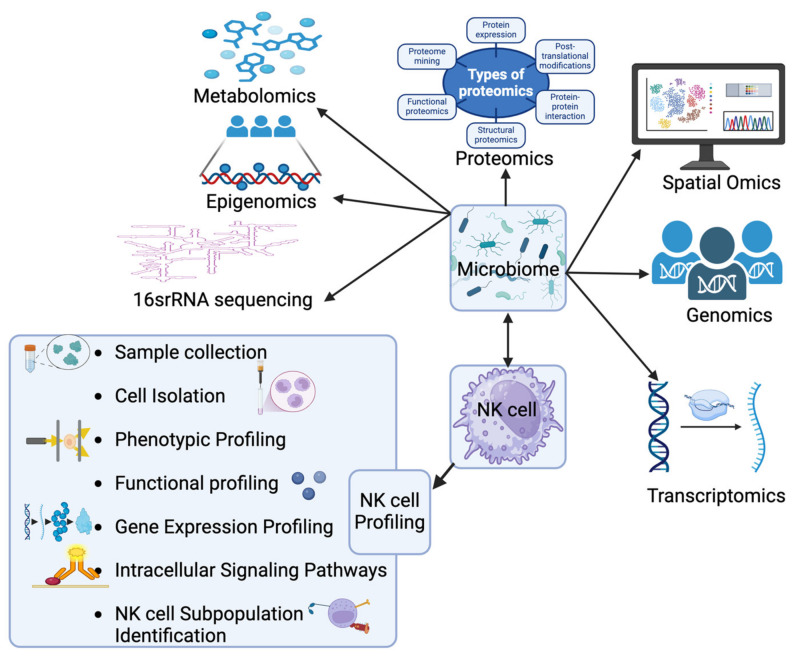
Integration of microbiome profiling and NK cell analysis as unified biomarkers for managing PDAC. This figure illustrates a suggested comprehensive diagnostic approach for identifying unified biomarkers to predict treatment resistance and side effects in pancreatic ductal adenocarcinoma (PDAC) patients. The approach integrates microbiota analysis from oral washing samples, stool, or tumor tissues using omics technologies, such as 16S rRNA sequencing, with NK cell profiling. 16S rRNA sequencing is a genomic technique that profiles microbial communities by analyzing the 16S ribosomal RNA gene, providing insights into microbial diversity and how the microbiome may influence PDAC treatment outcomes. The analysis can be performed in PDAC patient cohorts, categorized by long vs. short overall survival, responders vs. non-responders, and patients with vs. without side effects. By combining microbiome data with NK cell profiling, the goal is to identify combined biomarker signatures that optimize clinical outcomes. Additionally, the figure highlights the importance of metagenomics and metaproteomics, which offer complementary insights into the interactions between patient cells and the microbiome, essential for developing robust biomarkers for personalized PDAC therapies. NK cell profiling involves isolating NK cells from patient samples (e.g., peripheral blood, tumor tissues) and analyzing their phenotype, function, and gene expression. This includes flow cytometry to identify NK cell subpopulations (e.g., CD56^dim^CD56^bright^), functional assays (e.g., cytotoxicity and cytokine production), and gene expression profiling (via RNA sequencing or qRT-PCR). These analyses reveal NK cell activation, exhaustion, or senescence, helping to identify biomarkers associated with treatment resistance and side effects, thereby contributing to more personalized PDAC therapies.

Understanding how microbiota interact with NK cells could provide key insights into treatment toxicity and efficacy (Figure 4). Identifying microbial signatures linked to treatment resistance and side effects may guide personalized therapies, ultimately improving patient outcomes. Therefore, the integration of non-invasive diagnostic tools, such as oral mucosa smears and stool samples, could offer a practical means to monitor microbiome composition and NK cell function [3,4,17,25,29]. This combined approach could help identify unified microbial–NK cell signatures associated with treatment efficacy, failure, and side effects. Moreover, microbiome-targeting interventions, such as probiotics, synbiotics, antibiotics, and FMT, may be less invasive than direct NK cell augmentation strategies. These interventions could restore microbial balance and counteract the three main mechanisms of NK cell suppression (low NK cell proliferation, impaired cytotoxicity, and reduced tumor infiltration). By addressing these mechanisms, the interventions could overcome PDAC treatment resistance using the ‘triple NK cell biomarker approach’, which targets the tumor, microbiota, and NK cell function [175].

The complexity of microbiome–host interactions require a combined biomarker approach, integrating microbiome and NK cell phenotyping to more accurately predict PDAC treatment outcomes. We hypothesize that changes in NK cell phenotype result from microbiome dysbiosis, and this needs to be validated. If confirmed, targeting the microbiome to modify NK cell activity could be a potential therapeutic strategy.

Microbiome-targeting interventions, including probiotics, synbiotics, antibiotics, and FMT, have the potential to enhance NK cell activation, restore microbial balance, and alleviate treatment-related side effects [125,135,136,137]. For example, FMT from long-term PDAC survivors could shift the gut microbiota towards a healthier state, potentially supporting immune function and improving treatment outcomes [15]. Antibiotics, on the other hand, can affect microbial populations, potentially altering inflammation and impacting the effectiveness of concurrent cancer therapies or their side effects [66,72,155,156,157,158,159,160].

Several challenges remain in translating these findings into clinical practice, including individual variability in microbiota composition and difficulties in applying preclinical data to human cancer treatment. More research is needed to fully understand how dysbiosis contributes to NK cell dysfunction and treatment-related outcomes in PDAC. A promising approach is the use of non-invasive tools to monitor microbiome and NK cell phenotypes in PDAC patients. Oral mucosa smears and stool samples could be utilized to profile microbial communities and assess NK cell function, helping to identify microbial signatures linked to treatment response and treatment-related side effects. Tracking microbiota changes over time may also reveal dysbiosis patterns associated with treatment resistance or side effects.

However, one key challenge is establishing baseline microbiota profiles that distinguish normal microbial composition from cancer-associated dysbiosis. Comparative studies between healthy individuals and PDAC patients, responders versus non-responders, patients with long overall survival versus short overall survival, and those with and without side effects could identify early biomarkers of dysbiosis, leading to targeted microbiome interventions that improve treatment outcomes in PDAC. Integrating NK cell profiling with microbiome analysis overcomes the limitations of single-biomarker approaches, providing a more holistic view of the patient’s immune and microbial landscape. This integrated approach could also reduce the risks associated with microbiome-modifying therapies, such as antibiotic resistance or unintended microbial shifts, offering a better model for predicting treatment response and side effects.

Currently, there are no clinical trials specifically investigating the combination of NK cell-derived therapeutic modalities and dysbiosis-targeted therapies in PDAC. However, both approaches are under separate investigation for their potential in enhancing PDAC treatment. It is important, prospectively, to combine these two therapeutic modalities to maximize their potential synergistic effects. By addressing both dysbiosis and other tumor/TME factors related to induced NK cell suppression, we could provide a more holistic approach to overcoming treatment resistance in PDAC.

Future research should focus on identifying specific microbial signatures that influence NK cell activity in PDAC. Investigating these signatures could uncover novel therapeutic targets aimed at enhancing immune responses and overcoming treatment resistance. Clinical trials validating microbiome-targeting therapies—such as prebiotics, antibiotics, and FMT—are critical to assess their safety, efficacy, and potential to improve PDAC treatment outcomes and survival. Additional research in PDAC should involve simultaneous assessments of the microbiome in the oral cavity, gastrointestinal tract, bile duct, and tumor tissue, along with immune cell dynamics, specifically NK cell profiling, in blood and the TME. Understanding these interactions could clarify how microbial ecosystems and NK cells influence the TME, identifying new biomarkers and therapeutic targets to improve patient outcomes. A promising approach would be to develop a microbiome signature from noninvasive samples as a surrogate for intratumoral and TME profiles, combined with a circulatory NK cell signature as a proxy for NK cell dynamics in the tumor/TME. This integrated strategy represents a favorable direction for further research.

## 5. Conclusions

Targeting dysbiosis in PDAC offers considerable potential for overcoming treatment resistance and managing treatment-related side effects. By modulating the microbiome and enhancing NK cell function, we can potentially improve patient outcomes and quality of life. Future research integrating NK cell phenotype profiling with microbiome diagnostics could lead to personalized treatment strategies, offering more effective and less toxic therapies for PDAC patients. Addressing the challenges of establishing baseline microbiota profiles, identifying predictive microbial signatures, and validating microbiome-targeting therapies—combined with NK cell profiling to create a unified biomarker signature—could transform PDAC management. This approach would provide safer, more tailored therapeutic options [Table 1] for patients, improving both treatment efficacy and overall outcomes.

## Figures and Tables

**Table 1:** Association between microbiomes and NK cell function: clinical application.

Taxonomy	Role /Effect on NK Cells	Translational Clinical Application/Suggestion
Actinobacteria (phylum)	Protective in the oral microbiome. Carcinogenic in the gut microbiome) [51,66,85].	Phylum regulation in the oral and gut microbiomes through direct or indirect dysbiosis-targeting strategies.
Acidovorax Ebreus; A. ebreus (species, genus: Acidovorax, phylum: proteobac)	Carcinogenic by reducing immune cells [4,10,18,19,20].	Species depletion through direct or indirect dysbiosis-targeting strategies.
Acinobacter baumannii (species; genus: Acinobacter, phylum: Proteobacteria)	Carcinogenic in the intratumor microbiome [51,66].	Intra-tumor species depletion through direct or indirect dysbiosis-targeting strategies.
Actinomyces (genus; phylum: Actinobacteria)	Carcinogenic in the oral and gut microbiome [20,85,103]. Negative prognostic marker in the intratumor microbiome [16].	Genus depletion in the intra-tumoral, intra-gut, and oral microbiomes through direct or indirect dysbiosis-targeting strategies.
Aggregatibacter (genus; phylum: Proteobacteria)	Negative prognostic marker in the intratumor microbiome [16].	Intra-tumor genus depletion through direct or indirect dysbiosis-targeting strategies.
Aggregatibacter actinomycetemcomitans (species; genus: Aggregatibacter phylum: proteobacteria)	Carcinogenic in the oral microbiome by promoting tumor favoring inflammation and NK cell exhaustion [4,10,16,18,19,20,29,51]	Species depletion in the oral microbiomes through direct or indirect dysbiosis-targeting strategies.
Akkermansia muciniphila (species; genus: Akkermansia, phylum: verrucomicrobia)	Carcinogenic in the intratumor microbiome. Protective in the gut microbiome by decreasing NK cell exhaustion and positive predictive marker for immunotherapy [4,10,16,18,19,20,147].	Increasing species abundance, for example, by prescribing NGPs as adjuvants for immunotherapy to enhance the efficacy of PD-1 inhibitors [132,133].
Alistipes shahii (species; genus: Alistipes, phylum: Bacteriodetes)	Carcinogenic in the gut microbiome [6].	Species depletion in the gut microbiome through direct or indirect dysbiosis-targeting strategies.
Alloprevotella (genus; phylum: Firmicutes)	Protective in the oral microbiome [103].	Increasing genus abundance, for example, by prescribing NGPs, probiotics, lifestyle changes, vitamin therapy.
Alloscardovia omnicolens (species; genus: Alloscardovia, phylum: Actinobacteria)	Carcinogenic in the gut microbiome [147].	Species depletion in the gut microbiome through direct or indirect dysbiosis-targeting strategies.
Altidipes indistinctus (species; genus: Altidipes; phylum: Bacteroidetes)	Positive predictive marker for immunotherapy and response to IL-10+CpG oligonucleotide. Protective by decreasing NK cell exhaustion [4,10,16,17,18,19,20,132,133].	Increasing species abundance, for example, by prescribing NGPs, probiotics, lifestyle changes, vitamin therapy or as adjuvants for immunotherapy or IL-10+CpG oligonucleotide to enhance the efficacy.
Alternaria alternata (species; genus: Alternaria, phylum: Ascomycota)	Carcinogenic by enhancing tumor favoring immunity, inducing NK cell exhaustion [4,10,18,19,20].	Species depletion through direct or indirect dysbiosis-targeting strategies.
Anaerostipes (genus; phylum: Firmicutes)	Protective in the gut microbiome [8,108].	Increasing genus abundance, for example, by prescribing NGPs, probiotics, FM, lifestyle changes, vitamin therapy.
Atopobium parvulum (species; genus: Atopobium; phylum: Actinobacteria)	Carcinogenic in the oral microbiome [38].	Species depletion in the oral microbiomes through direct or indirect dysbiosis-targeting strategies.
Bacidiomycota (phylum of fungi)	Carcinogenic by activating mannose binding lectin, suppressing NK cells cytotoxicity [83].	Phylum depletion through direct or indirect dysbiosis-targeting strategies.
Bacillus clausii (species; genus: Bacillus, phylum: Firmicutes)	Protective in the intratumor microbiome [15].	Increasing species abundance, for example, by prescribing tumor-colonizing bacteria, oral probiotics, FM, lifestyle changes, vitamin therapy.
Bacteroidales (order; phylum: Bacteroidetes)	Carcinogenic in the gut microbiome [108].	Order depletion in the gut microbiome through direct or indirect dysbiosis-targeting strategies.
Bacteroides (genus; phylum: bacteroidetes)	Carcinogenic in the oral, gut and intratumor microbiome [104,147]. PIPN inducer, activating Astrocytes and TLR4/p38MAPK Pathway, with effects on NK cell regulation [6,96,97].	Genus depletion in the intra-tumoral, intra-gut, and oral microbiomes through direct or indirect dysbiosis-targeting strategies.
Bacteroides coprocola (species; genus: Bacteroides, phylum: Bacteroidetes)	Protective in the gut microbiome [147].	Increasing species abundance in the gut for example, by prescribing oral probiotics, FMT, lifestyle changes, vitamin therapy.
Bacteroidetes (phylum)	Carcinogenic in the gut microbiome [51].	Phylum depletion in the gut microbiome through direct or indirect dysbiosis-targeting strategies.
Bifidobacterium (genus; phylum: Actinobacteria)	Protective in the gut microbiome [8]. It can increase NKA [2,63] and acts as a positive predictive biomarker for immunotherapy to potentiate the efficacy of immune checkpoint inhibitors [62,63,133].	Increasing genus abundance in the gut microbiome, for example, by prescribing FMT, NGPs, probiotics, lifestyle changes, vitamin therapy or as adjuvants for immunotherapy to potentiate its effect.
Bifidobacterium bifidum (species; genus: Bifidobacterium, phylum: Actinobacteria)	Protective in the gut microbiome [147].	Increasing genus abundance in the gut microbiome, for example, by prescribing FMT, NGPs, probiotics, postbiotics, lifestyle changes, vitamin therapy.
Bifidobacterium pseudocatenulatum (species; genus: Bifidobacterium, phylum: Actinobacteria)	Protective in the gut microbiome [6].	Increasing species abundance in the gut microbiome, for example, by prescribing FMT, NGPs, probiotics, postbiotics, lifestyle changes, vitamin therapy.
Blautia (genus; phylum: Firmicutes)	Protective in the gut microbiome [8].	Increasing species abundance in the gut microbiome, for example, by prescribing FMT, NGPs, probiotics, postbiotics, lifestyle changes, vitamin therapy.
Blautia obeum (species; genus; phylum: Firmicutes)	Protective in the gut microbiome [6].	Increasing species abundance in the gut microbiome, for example, by prescribing FMT, NGPs, probiotics, postbiotics, lifestyle changes, vitamin therapy.
Bradyrhizobium (genus; phylum: Proteobacteria)	Positive prognostic marker in the intratumor microbiome [16].	Increasing genus abundance in the intratumor microbiome, for example, by prescribing tumor-colonizing bacteria, FMT, NGPs, probiotics, postbiotics, lifestyle changes, vitamin therapy.
Capnocytophaga (genus; phylum: Bacteroidetes)	Protective in the oral microbiome [20].	Increasing genus abundance in the gut microbiome, for example, by prescribing NGPs, probiotics, postbiotics, lifestyle changes, vitamin therapy.
Cardiobacterium (genus; phylum: Proteobacterium)	Carcinogenic in the gut microbiome [51].	Genus depletion in the gut microbiome through direct or indirect dysbiosis-targeting strategies.
Citrobacter (genus; phylum: Protecobacteria)	Carcinogenic in the intratumor microbiome [65].	Genus depletion in the intratumor microbiome through direct or indirect dysbiosis-targeting strategies.
Citrobacter freundii (species; genus: Citrobacter, phylum: proteobacteria)	Carcinogenic in the intratumor microbiome, contributing to immunosuppression, upregulation of oncogenic pathways, and downregulation of tumor suppressive pathways [3,19,54,103].	Species depletion in the intra-tumoral, through direct or indirect dysbiosis-targeting strategies.
Cladocopium symbiosum (species; genus: Cladocopium, phylum: Apicomplexa)	Carcinogenic in the gut microbiome [6].	Species depletion in the gut microbiome through direct or indirect dysbiosis-targeting strategies.
Clostridiaceae (family; phylum: Firmicutes)	Protective in the gut microbiome [15].	Increasing family abundance in the gut microbiome, for example, by prescribing FMT, NGPs, probiotics, postbiotics, lifestyle changes, vitamin therapy and so on.
Clostridiales (order; phylum: Firmicutes)	Protective in the gut microbiome [108].	Increasing order abundance in the gut microbiome, for example, by prescribing FMT, NGPs, probiotics, postbiotics, lifestyle changes, vitamin therapy.
Clostridium (genus; phylum: Firmicutes)	Protective in the gut microbiome, [108] negative prognostics in gut and oral sample [15,17] but protective intratumorally [146].	Reducing abundance in gut and oral samples using probiotics, while increasing intratumoral abundance with tumor-colonizing bacteria
Clostridium IV (group; genus: Clostridium, phylum: Firmicutes)	Protective in the gut microbiome [8].	Increasing species abundance in the gut microbiome, for example, by prescribing FMT, NGPs, probiotics, postbiotics, lifestyle changes, vitamin therapy.
Clostridium bolteae (species; genus: Clostridium, phylum: Firmicutes)	Carcinogenic in the gut microbiome [6].	Species depletion in the gut microbiome through direct or indirect dysbiosis-targeting strategies.
Clostridium_sensu stricto 1 (species; genus: Clostridiumphylum: Firmicutes)	Negative prognostic marker in the intratumor microbiome [16]; PIPN preventive in the gut microbiome, interfering with astrocytes and the TLR4/p38MAPK pathway [96,97].	Increasing species abundance in the gut through FMT to reduce PIPN, while promoting intratumoral abundance with tumor-colonizing bacteria
Collinsella aerofaciens (species; genus: Collnisella, phylum: Actinobacteria)	Protective in the gut microbiome [6].	Increasing species abundance in the gut microbiome through direct or indirect dysbiosis-targeting strategies.
Corynebacterium (genus; phylum: Actinobacteria)	PIPN preventive, by interfering with astrocytes and the TLR4/p38MAPK pathway [96,97].	Increasing genus abundance in the gut through FMT to reduce PIPN, while promoting intratumoral abundance with tumor-colonizing bacteria.
Coprococcus (genus; phylum: Firmicutes)	Protective in the gut microbiome [6,8].	Increasing genus abundance in the gut microbiome through direct or indirect dysbiosis-targeting strategies.
Desulfovibrio (genus; phylum: Proteobacteria)	Positive prognostic marker in the intratumor microbiome [16].	Increasing intratumoral abundance through direct or indirect dysbiosis-targeting strategies, such as using tumor-colonizing bacteria.
Dialister (genus; phylum: Firmicutes)	Carcinogenic in the oral microbiome [85].	Reducing genus abundance in the oral microbiome using direct and indirect dysbiosis-targeting strategies.
Escherichia coli (species; genus: Escherichia, Phylum: Proteobacteria)	Carcinogenic in the oral microbiome [20], decreasing NK cell cytotoxicity [92], and inducing diarrhea by reducing IL-22 production in NK cells, which normally protect the integrity of the intestinal epithelial barrier damaged by enterotoxigenic Escherichia coli. Negative predictive marker for gemcitabine response [46,66].	Reducing species abundance in the oral microbiome using direct and indirect dysbiosis-targeting strategies. For example, Lactobacillus probiotics can be used to increase gemcitabine efficacy and prevent diarrhea
Elizabethkingia (genus; phylum: Bacteroidetes)	Carcinogenic [54].	Genus depletion through direct or indirect dysbiosis-targeting strategies.
Enhydrobacter (genus; phylum: Proteobacterai)	Positive prognostic marker in the intratumor microbiome [16].	Increasing intratumoral abundance through direct or indirect dysbiosis-targeting strategies, such as using tumor-colonizing bacteria.
Enterobacter (genus; phylum: Proteocteria)	Carcinogenic in the gut microbiome [8]; protective intratumorally, with TIGIT upregulation [31,32].	Genus depletion through direct and indirect dysbiosis-targeting strategies. Increasing intratumoral abundance using direct or indirect dysbiosis-targeting approaches, such as tumor-colonizing bacteria.
Enterobacter asburiae (species; genus: Enterobacter, phylum: Poteobacteria)	Carcinogenic in the gut and intratumor microbiome [28,103].	Species depletion in the gut and intra-tumor microbiomes through direct or indirect dysbiosis-targeting strategies.
Enterobacteriaceae (family; phylum: Proteobacteria)	Carcinogenic in the oral and intratumor microbiome [20,56,66].	Species depletion in the gut and intra-tumor microbiomes through direct or indirect dysbiosis-targeting strategies.
Enterococcus hirae (species; genus: Enterococcus, phylum: Firmicutes)	Positive predictive for immunotherapy response [62].	Increasing species abundance through direct or indirect dysbiosis-targeting strategies or prescribing the species as a probiotic product to be used as adjuvant therapy for immunotherapy.
Erysipelotrichaeceae (family; phylum: Firmicutes)	Protective in the gut microbiome [6].	Increasing family abundance in the gut microbiome through direct or indirect dysbiosis-targeting strategies, such as using FMT.
Eubacterium hallii (speies; genus: Eubacterium, phylum: Firmicutes)	Protective in the gut microbiome [6].	Increasing species abundance in the gut microbiome through direct or indirect dysbiosis-targeting strategies, such as using FMT.
Eubacterium ventriosum (species; genus: Eubacterium, phylum: Firmicutes)	Protective in the gut microbiome [85].	Increasing species abundance in the gut microbiome through direct or indirect dysbiosis-targeting strategies, such as using FMT.
Faecalibacterium (genus; phylum: Firmicutes)	Protective in the gut microbiome [108].	Increasing species abundance in the gut microbiome through direct or indirect dysbiosis-targeting strategies, such as using FMT.
Faecalibacterium prausnitzii (species; genus: Faecalibacterium; phylum: Firmicutes)	Protective in the gut microbiome [85,147]	Increasing species abundance in the gut microbiome through direct or indirect dysbiosis-targeting strategies, such as using FMT.
Firmicutes (phylum)	Protective in the gut microbiota [149], reducing bile acid production and bile acid induced carcinogenesis, while increasing NK cell activity through bile acid reduction and butyrate production [72,85,102,109,144]	Phylum regulation in the gut and intra-tumor microbiomes through direct or indirect dysbiosis-targeting strategies
Flavobacterium (genus; phylum: Bacteroidetes)	Positive prognostic marker in the intratumor microbiome [16].	Increasing genus abundance in the intra-tumor microbiome through direct or indirect dysbiosis-targeting strategies, such as tumor-colonizing bacteria.
Flavonifractor (genus; phylum: Firmicutes)	Protective in the gut microbiome [8].	Increasing genus abundance in the gut microbiome through direct or indirect dysbiosis-targeting strategies, such as FMT.
Fusobacterium (genus; Fusobacteria)	Carcinogenic in the oral microbiome [104].	Species depletion in the oral microbiome through direct or indirect dysbiosis-targeting strategies.
Fusobacterium nucleatum (species; genus: Fusobacterium, phylum: Fusobacteria)	Carcinogenic in the gut and intratumor microbiome via cytokine secretion and decreased NK cell tumor infiltration, serving as a negative prognostic marker intratumorally [19,27,28,29,73,103,106,147].	To decrease the species using probiotics to optimize clinical outcomes, particularly to potentiate the effects of 5-FU and oxaliplatin.
Gammaproteobacteria (class; phylum: Proteobacteria)	Carcinogenic in the intratumor microbiome [66], a negative predicative marker for gemcitabine response [28,65,66] due to bacterial CDA, and for immunotherapy, while serving as a positive predicative marker for chemotherapy (e.g., 5-FU and oxaliplatin); immune regulation via CDA [101,103,104].	To be prescribed as tumor-colonizing bacteria alongside oxaliplatin and 5-FU-based chemotherapy to potentiate their effects. Probiotics can be used to reduce species abundance when gemcitabine is prescribed.
Granulicatella adiacens (species; genus: Granulicatella, Firmicutes)	Carcinogenic in the oral microbiome [38].	Species depletion in the oral microbiome through direct or indirect dysbiosis-targeting strategies.
Haemophilus abundance (species; genus: Haemophilus, phylum: Proteobacterai)	Carcinogenic in the oral microbiome [56].	Species depletion in the oral microbiome through direct or indirect dysbiosis-targeting strategies.
Helicobacter hepaticus (species; genus: Helicobacter, phylum: Proteobacteria)	Carcinogen (seroprevalence), protective intratumorally, increasing NK cell tumor infiltration [28,29].	Patients who are seropositive for *H. hepaticus* have a higher risk of progression and may benefit from receiving the species as tumor-colonizing bacteria to induce NK cell infiltration.
Helicobacter pylori (species; genus: Helicobacter, phylum: Proteobacteria)	Carcinogenic, with NK cell suppression [123,124].	Eradicating *H. pylori* using antibiotics or probiotics. Alternatively, using Interleukin 12 in patients with dysbiosis positive for *H. pylori* to induce gamma interferon activation and enhance NK cell activity.
Herbaspirillum (genus; phylum: Proteobacteria)	Carcinogenic in the intratumor microbiome [49].	Genus depletion in the intra-tumor microbiome through direct or indirect dysbiosis-targeting strategies.
Klebsiella (genus; phylum: Proteobacteria)	Carcinogenic in the gut and intratumor microbiome [8,51,66].	Genus depletion in the gut and intra-tumor microbiome through direct or indirect dysbiosis-targeting strategies.
Klebsiella pneumoniae (species; genus: Proteobacteria, phylum: proteobacteria)	Carcinogenic in the gut and intratumor microbiome [19,103], leading to NK cell suppression [6,124].	Species depletion in the gut and intra-tumor microbiome through direct or indirect dysbiosis-targeting strategies.
Lachnospiraceae (family; phylum: Firmicutes)	Protective in the gut microbiome in some studies [6,85], but carcinogenic in the oral and gut microbiome, reported by another study [56,108].	Further investigation is needed to specify the strain-level effects and determine the clinical relevance, as well as to regulate dysbiosis accordingly.
Lactobacillus (genus; phylum: Firmicutes)	Carcinogenic in the oral and gut microbiome [6,103].	Further investigation is needed to specify the strain-level effects and determine the clinical relevance, as well as to regulate dysbiosis accordingly.
Lactobacillus plantarum (species; genus: Lactobacillus, phylum: Firmicutes)	Protective in the gut microbiome, while carcinogenic in the intratumor microbiome [147].	Increasing genus in the gut microbiome and reducing intratumoral abundance through direct and indirect dysbiosis-targeting strategies.
Leptotrichia (genus; phylum: Fusobacteria)	Carcinogenic in the oral microbiome [103]; however, protective in both the gut and oral microbiome, according to other studies [20,104].	Further investigation is needed to specify the strain-level effects and determine the clinical relevance, as well as to regulate dysbiosis accordingly.
Listeria whelshimer I Serover 6B SLCC 5334 (strain; genus: Listeria, phylum: Firmicutes)	Protective, inducing IL-12 production and NK cell activity via LPS [146,153,169].	It can be used as tumor-colonizing bacteria or as a vaccination to target tumor and enhance the response to gemcitabine.
Malassezia globosa (species of yeast; genus: Malassezia, phylum: Ascomycota)	Carcinogen, enhancing tumor favoring immunity by suppress NK cells through the release of C3a and C5a, thereby reducing NKG2D [161].	Species depletion using antifungal drugs or other direct and indirect dysbiosis-targeting modalities
Megamonas (genus; phylum: Firmicutes)	Protective in the gut microbiome [51]; positive prognostic marker in the intratumor microbiome [16].	Increasing genus abundance in the gut and intra-tumor microbiomes through direct or indirect dysbiosis-targeting strategies, such as tumor-colonizing bacteria.
Megasphaera (genus; phylum: Firmicutes)	Protective and a positive prognostic marker in the intratumor microbiome [15,16,104], while carcinogenic in the oral microbiome [104].	Increasing genus abundance in the intratumoral microbiome through strategies like tumor-colonizing bacteria, and depleting genus abundance in the oral microbiome using appropriate dysbiosis-targeting approaches.
Mycobacterium (genus; phylum: Actinobacteria)	Protective through NKp44 interaction [45,47].	Increasing genus abundance using direct and indirect dysbiosis-modulating approach.
Mycoplasma hyorhinis (species; genus: Mycoplasma, phylum: Mycoplasmatota)	Negative predictive marker for gemcitabine due to decreased cytotoxicity caused by bacterial CDA [28,65].	Decreasing species abundance using direct and indirect dysbiosis-modulating modalities, such as antibiotics and vaccination, to prevent gemcitabine resistance.
Neisseria (genus; phylum: Proteobacteria)	Protective in the oral microbiome [104] and a negative prognostic marker in the intratumor microbiome [16].	Decreasing genus abundance in the intratumoral microbiome and increasing genus abundance in the oral microbiome using direct and indirect dysbiosis-modulating modalities.
Neisseria elongata (species; genus: Neisseria, phylum: Proteobacteria)	Protective in the oral microbiome [20,38,103,104].	Increasing species abundance in the oral microbiome through direct or indirect dysbiosis-targeting strategies.
Odoribacter (genus; phylum: Bacteroidetes)	Carcinogenic in the gut microbiome [108].	Genus depletion in the gut microbiome using direct or indirect dysbiosis-targeting approach.
Parabacteroides (genus; phylum: Bacteroidetes)	Carcinogenic in the gut microbiome [6,51].	Genus depletion in the gut microbiome by direct or indirect dysbiosis-targeting.
Propionibacterium acnes (species; genus: Propionibacterium, phylum: Actinobacteria)	Carcinogenic, through cytokine modulation and Hedgehog signaling activation [21,22,23].	Species depletion using, for example, antibiotics, bacteriophages, or other modalities to target dysbiosis.
Porphyromonas (genus; phylum: Bacteroidetes)	Protective in the oral microbiome [20] and a negative prognostic marker in the intratumor microbiome [16].	Increasing genus in the oral microbiome and reducing intratumoral abundance through direct and indirect dysbiosis-targeting strategies.
Prophyromonas gingivalis (species; genus: Prophyromonas phylum: bacteroidetes)	Carcinogen in the oral microbiome, inducing tumor-favoring inflammation and NET induced NK cell suppression; direct effect on NK cells has not been investigated [9,16,17,20,40].	Species depletion using, for example, antibiotics or other modalities to target dysbiosis.
Proteobacteria (phylum)	Carcinogenic in the gut and intratumor microbiomes, promoting tumor metastasis, bile acid induced carcinogenesis, and immunosuppression [9,16,17,18,19,30,37,40,51,103].	This phylum needs to be analyzed at the strain level, as generalizing clinical outcomes at the phylum level is not applicable due to controversial results.
Prevotella (genus; phylum: Bacteroidetes)	Carcinogenic in the oral microbiome [103], but protective in both oral and gut microbiomes [20,51].	Further investigation is needed to specify the species-level effects and determine the clinical relevance, as well as to regulate dysbiosis accordingly.
Prevotella pallens (species; genus: Prevotella, phylum: Bacteroidetes)	Carcinogenic in the oral microbiome [85].	Species depletion in the oral microbiome using modalities to target dysbiosis.
Prevotella sp. C561(strain; species P.sp, genus: Prevotella, phylum: Bacteroidetes)	Carcinogenic in the oral microbiome [85].	Strain depletion in the oral microbiome using modalities to target dysbiosis.
Pseudomonas (genus; phylum: Proteobacteria)	Carcinogen in the intratumor microbiome [45,47,49,150].	Genus depletion in the intra-tumor microbiome through direct or indirect dysbiosis-targeting strategies.
Pseudomonadaceae (family; phylum: Proteobacteria)	Carcinogenic in the intratumor microbiome [65].	Family depletion in the intra-tumor microbiome through direct or indirect dysbiosis-targeting strategies; however, further investigation at the species level is recommended.
Pseudomonas aeroginosa (species; genus: Pseudomonas, phylum: Proteobacteria)	Carcinogenic [45,47,150].	Species depletion in the intra-tumor microbiome through direct or indirect dysbiosis-targeting strategies.
Pseudoxanthomonas (genus; phylum: Proteobacteria)	Protective as the intratumor microbiome, triggering antitumor immunity and serving as a positive prognostic marker [12,15].	Increasing genus abundance in the intratumoral microbiome through strategies like tumor-colonizing bacteria or other appropriate dysbiosis-targeting approaches.
Romboutsia timonensis (species; genus: Romboutsia, Phylum: Firmicutes)	Protective in the gut microbiome [147].	Increasing species abundance in the gut microbiome through strategies like FMT or other appropriate dysbiosis-targeting approaches.
Rothia (genus; phylum: Actinobacteria)	Carcinogenic in the oral microbiome [20].	Genus depletion in the oral microbiome by targeting dysbiosis appropriately.
Ruminococcaceae (family; phylum; Firmicutes)	Protective in the gut microbiome [108].	Increasing family abundance in the gut microbiome through strategies like FMT or other appropriate dysbiosis-targeting approaches. However, further investigation at genus or species level is recommended.
Ruminococcus (genus; phylum: Firmicutes)	Positive predictive marker for immunotherapy and response to IL-10+CpG oligonucleotide [28,62,130,155].	To optimize clinical outcomes, using related targeting products to increase genus abundance and enhance tumor response to IL-10 + CpG oligodeoxynucleotide.
Streptococcus sanguinis (species; genus: Streptococcus, phylum: Firmicutes)	Protective in the gut microbiome [6].	Increasing species abundance in the gut microbiome through strategies like FMT or other appropriate dysbiosis-targeting approaches.
Saccharmycetes (class; phylum: Ascomycota)	Protective, triggering antitumor immunity and serving as a positive prognostic marker [15].	Increasing class abundance through appropriate dysbiosis-targeting approaches. However, further investigation at genus or species level is recommended.
Saccharopolyspora (genus; Phylum: Actinobacteria)	Protective and positive prognostic marker in the intratumor microbiome) [15]	Increasing genus abundance in the intratumoral microbiome through strategies like tumor-colonizing bacteria or other appropriate dysbiosis-targeting approaches.
Salmonella (genus; phylum: Proteobacteria)	Protective in the intratumor microbiome, activating the inflammatory cytokine interleukin-1beta and promoting NK cell activation [154].	Increasing genus abundance in the intratumoral microbiome through strategies like tumor-colonizing bacteria or other appropriate dysbiosis-targeting approaches.
Selenomonasb (genus; phylum: Firmicutes)	Carcinogenic in the gut microbiome [8], but protective in the oral microbiome [20].	Genus depletion in the gut microbiome, with an increase in the oral microbiome, using appropriate dysbiosis-targeting strategies. However, further investigation at the strain level is recommended.
Shigella sonnei (species; genus: Shigella, phylum: Proteobacteria)	Carcinogenic due to upregulation of oncogenic pathways, immunosuppression, and TME reprogramming [17].	Species depletion: however, further investigation at the strain level, particularly regarding oral-gut-intratumoral interactions and their clinical effects, is recommended.
Solobacterium (genus; phylum: Firmicutes)	Carcinogenic in the oral microbiome [85].	Genus depletion in the oral microbiome by targeting dysbiosis appropriately.
Sphingomonas (genus; phylum: Proteobacteria)	Carcinogenic in the intratumor microbiome [49]; protective and a positive prognostic marker in the gut microbiome [15,16].	Genus depletion in the intra-tumor microbiome and increasing in the gut microbiome by targeting dysbiosis appropriately.
Spirochaeta (genus; phylum: Spirochaetes)	Protective and positive prognostic marker in the intratumor microbiome [15,16], but carcinogenic in the oral microbiome [104].	Genus depletion in the oral microbiome, with an increase in the intratumor microbiome, using appropriate dysbiosis-targeting strategies.
Streptococcus (genus; phylum: Firmicutes)	Carcinogenic in the oral, intratumor and gut microbiomes [20,65].	Genus depletion in the oral, intratumor and gut microbiome, using appropriate dysbiosis-targeting strategies. However, further investigation at species and strain level is recommended.
Streptococcus mitis (species; genus: Streptococcus, phylum: Firmicutes)	Protective in the oral microbiome [20].	Increasing genus abundance in the oral microbiome through appropriate dysbiosis-targeting approaches.
Streptococcus anginosus (sepecies; genus: Streptococcus, phylum: Firmicutes)	Carcinogenic in the gut microbiome [85].	Species depletion in the gut microbiome, using appropriate dysbiosis-targeting strategies.
Streptococcus australis (sepecies; genus: Streptococcus, phylum: Firmicutes)	Protective in the oral microbiome [85].	Increasing species abundance in the oral microbiome through appropriate dysbiosis-targeting approaches.
Streptococcus mutans (sepecies; genus: Streptococcus, phylum: Firmicutes)	Carcinogenic in the gut microbiome [6].	Species depletion in the gut microbiome, using appropriate dysbiosis-targeting strategies.
Streptococcus oralis (sepecies; genus: Streptococcus, phylum: Firmicutes)	Carcinogenic in the gut microbiome [85].	Species depletion in the gut microbiome, using appropriate dysbiosis-targeting strategies.
Streptococcus salivarius (sepecies; genus: Streptococcus, phylum: Firmicutes)	Protective in the oral microbiome [85].	Increasing species abundance in the oral microbiome through appropriate dysbiosis-targeting approaches.
Streptococcus thermophilus (sepecies; genus: Streptococcus, phylum: Firmicutes)	Protective in the oral microbiome [85].	Increasing species abundance in the oral microbiome through appropriate dysbiosis-targeting approaches.
Streptococcus vestibularis(sepecies; genus: Streptococcus, phylum: Firmicutes)	Carcinogenic in the gut microbiome [85].	Species depletion in the gut microbiome, using appropriate dysbiosis-targeting strategies.
Streptomyces (genus; phylum: actinobacteria)	Protective in the intratumor microbiome, triggering antitumor immunity and NK cell infiltration [48]; positive prognostic marker [15].	Increasing genus abundance in the intratumoral microbiome through strategies like tumor-colonizing bacteria or other appropriate dysbiosis-targeting approaches.
Subdoligranulum (genus; phylum: Firmicutes)	Protective in the gut microbiome [108].	Increasing genus abundance in the gut microbiome through appropriate dysbiosis-targeting approaches.
Tannerella (genus; phylum: Bacteroidetes)	Protective in the oral microbiome [20].	Increasing genus abundance in the oral microbiome through appropriate dysbiosis-targeting approaches.
Treponema (genus; phylum: Spirochaetes)	Carcinogenic in the oral microbiome [104].	Genus depletion in the oral microbiome, using appropriate dysbiosis-targeting strategies.
Turicibacter (genus; phylum: Firmicutes)	PIPN preventive, interfering with astrocytes and TLR4/p38MAPK pathway, and regulating NK cell function [96,97,99].	The genus needs to be increased in the gut to reduce PIPN, using, for example, FMT or other appropriate approaches to target dysbiosis.
UCG-005 (genus, Phylum: Firmicutes)	PIPN Inducer, activating astrocytes and the TLR4/p38MAPK pathway, and regulating NK cell [96,97,99].	The genus needs to be decreased in the gut to reduce PIPN, using, for example, antibiotics, dietary changes, or other appropriate approaches to target dysbiosis.
Veillonella (genus; phylum: Firmicutes)	Carcinogenic in the gut microbiome [8,51].	Genus depletion in the gut microbiome, using appropriate dysbiosis-targeting strategies.
Veillonella atypica (species; genus: Veillonella, phylum: Firmicutes)	Carcinogenic in the gut microbiome [85,147].	Species depletion in the gut microbiome, using appropriate dysbiosis-targeting strategies.
Veillonella parvula (species; genus: Veillonella, phylum: Firmicutes)	Carcinogen in the gut microbiome [85].	Species depletion in the gut microbiome, using appropriate dysbiosis-targeting strategies.

This table provides an overview of findings from various studies regarding the role of specific microorganisms in the context of PDAC. The effect of microorganisms on NK cells is also mentioned where applicable, based on available studies; if no study addresses this, the corresponding field is left blank. It is important to note that there is ongoing debate regarding the classification of certain organisms as either ’protective’ or ’carcinogenic’. The effect of these organisms can vary depending on the specific environmental context in which they are found. While this table aims to summarize key results, we acknowledge that oversimplification may occur, and the complexity of these microbiome–tumor interactions should be considered.

## Data Availability

Data sharing is not applicable to this review article as it does not contain original datasets. All relevant information and sources are cited within the manuscript.

## Abbreviations

5-FU: 5-Fluorouracil; CDA: Cytidine Deaminase; CpG: Unmethylated cytosine-phosphate-guanine; FMT: Fecal microbiota Transplant; IL-10: Interleukin 10; MAPK: Mitogen-activated protein kinase; NET: Neutrophil extracellular traps; NGPs: Next-generation probiotics; NKA: Natural killer cell activity; OIPN: oxaliplatin-induced neuropathy; PD-1: Programmed Cell Death Protein 1; PIPN: paclitaxel-induced peripheral neuropathy; TLR4: Toll-like receptor 4.
